# Assessing comorbidity and correlates of wasting and stunting among children in Somalia using cross-sectional household surveys: 2007 to 2010

**DOI:** 10.1136/bmjopen-2015-009854

**Published:** 2016-03-09

**Authors:** Damaris K Kinyoki, Ngianga-Bakwin Kandala, Samuel O Manda, Elias T Krainski, Geir-Arne Fuglstad, Grainne M Moloney, James A Berkley, Abdisalan M Noor

**Affiliations:** 1 Spatial Health Metris Group, INFORM Project, Kenya Medical Research Institute/Wellcome Trust Research Programme, Nairobi, Kenya; 2 Warwick Medical School, Health Sciences Research Institute, University of Warwick, Warwick Evidence, Coventry, UK; 3 Department of Mathematics and Information sciences, Faculty of Engineering and Environment, Northumbria University, Newcastle upon Tyne, UK; 4 Department of Population Health, Luxembourg Institute of Health (LIH), Strassen, Luxembourg; 5 Biostatistics Research Unit, South African Medical Research Council, Pretoria, South Africa; 6 Division of Epidemiology and Biostatistics, School of Public Health, University of Witwatersrand, Johannesburg, South Africa; 7 Department of Mathematical Sciences, Norwegian University of Science and Technology, Trondheim, Norway; 8 Department of Statistics, Federal University of Paraná, Curitiba, Brazil; 9 Nutrition Section, United Nations Children's Fund (UNICEF), Kenya Country Office, UN Complex Gigiri, Nairobi, Kenya; 10 Kenya Medical Research Institute/Wellcome Trust Research Programme, Centre for Geographic Medicine Research (coast), Kilifi, Kenya; 11 Nuffield Department of Clinical Medicine, Centre for Tropical Medicine and Global Health, University of Oxford, Oxford, UK

**Keywords:** malnutrition, wasting, stunting, underweight, Geo-statistics, shared-component

## Abstract

**Objective:**

Wasting and stunting may occur together at the individual child level; however, their shared geographic distribution and correlates remain unexplored. Understanding shared and separate correlates may inform interventions. We aimed to assess the spatial codistribution of wasting, stunting and underweight and investigate their shared correlates among children aged 6–59 months in Somalia.

**Setting:**

Cross-sectional nutritional assessments surveys were conducted using structured interviews among communities in Somalia biannually from 2007 to 2010. A two-stage cluster sampling methodology was used to select children aged 6–59 months from households across three livelihood zones (pastoral, agropastoral and riverine). Using these data and environmental covariates, we implemented a multivariate spatial technique to estimate the codistribution and divergence of the risks and correlates of wasting and stunting at the 1×1 km spatial resolution.

**Participants:**

73 778 children aged 6–59 months from 1066 survey clusters in Somalia.

**Results:**

Observed pairwise child level empirical correlations were 0.30, 0.70 and 0.73 between weight-for-height and height-for-age; height-for-age and weight-for-age, and weight-for-height and weight-for-age, respectively. Access to foods with high protein content and vegetation cover, a proxy of rainfall or drought, were associated with lower risk of wasting and stunting. Age, gender, illness, access to carbohydrates and temperature were correlates of all three indicators. The spatial codistribution was highest between stunting and underweight with relative risk values ranging between 0.15 and 6.20, followed by wasting and underweight (range: 0.18–5.18) and lowest between wasting and stunting (range: 0.26–4.32).

**Conclusions:**

The determinants of wasting and stunting are largely shared, but their correlation is relatively variable in space. Significant hotspots of different forms of malnutrition occurred in the South Central regions of the country. Although nutrition response in Somalia has traditionally focused on wasting rather than stunting, integrated programming and interventions can effectively target both conditions to alleviate common risk factors.

**Strengths and limitations of this study**

- We examined the shared risk factors of wasting, stunting and determined the shared spatial distribution to show regions where the codistribution of the indicators is highly prevalent to inform policy development and implementation of interventions.
- Individual child level data improved the estimates in our analysis as it accounted for the variability between children ensuring more accurate estimates than by using aggregated data.
- Sustained conflict, which might exacerbate malnutrition in Somalia and continues to be the primary reason for displacement affecting the South Central zone, was not accounted for in this study.

## Introduction

Malnutrition is one of the major causes of childhood deaths in developing countries.^1^ ^2^ Globally, in 2011, 1 in 4 children (26%, 165 million) were stunted, 1 in 6 (16%, 101 million) were underweight, and 1 in 12 (8%, 52 million) were wasted. More than 90% of undernourished people live in developing countries.^3^ In Somalia, it was estimated that 35% of the population, approximately 2.85 million people, were affected by the food security crisis in 2011. In the Southern regions of the country, the prevalence of acute malnutrition was estimated to be at least 30%.^4^

Indicators of nutritional status include biomarkers, body composition analysis and simple anthropometry.^5^ Wasting (low weight-for-length/height) and stunting (low height-for-age) are the most commonly used indicators for individual assessment, designing programmes and assessing impact.^6^ ^7^ Stunting is thought to be an indicator of chronic or long-term nutritional inadequacy, while wasting is usually assumed to reflect an acute situation related to illness or lack of food.^7^ Wasting and stunting are normally presented as distinct nutritional problems, contributing separately to mortality and disease.^8^ A third indicator of malnutrition, underweight, defined as low weight-for-age, combines information on linear growth and bulk.^9^

Studies in developing countries have shown that wasting and stunting may be dependent on each other when compared to a standard population.^10^ ^11^ If linear growth falters due to infection or poor diet, catch-up growth might be attained once the infection is eliminated or the diet improves. However, in resource-poor settings, where dietary intake may be consistently inadequate or there is a high rate of infectious diseases, catch-up growth may be impossible, resulting in stunting.^11^ ^12^ In this context, wasting might precede linear growth retardation, and therefore it is possible that wasting directly influences linear growth;^13^ however, this is currently uncertain. In general terms, the worldwide variation of low weight-for-age and its age distribution are similar to those of low height-for-age.^14^

Owing to prolonged drought, famine and conflict in Somalia, the rates of malnutrition persistently remain at ‘critical’ levels and have been cited as among the highest in the world.^15^ ^16^ The distribution of malnutrition varies across space and time, influenced by climatic conditions and other factors with definable spatial and temporal dependencies.^17^ In Somalia, for example, conflict and drought lead to frequent displacement of vulnerable people leading to disruptions in livelihoods.^18^ Stunting and underweight may be affected by the cumulative effect of intermittent rates of wasting. Children presenting with multiple forms of malnutrition have been reported to be at a higher risk of mortality when compared to children with one form of malnutrition.^19^ This additive effect highlights the need to understand the common drivers and extent of coexistence of the indicators geographically in order to formulate effective strategies for intervention.

Previous analysis of the predictors of malnutrition in Somalia has focused on separate anthropometric measures, but not common correlates.^17^ In this study, we aimed to describe the spatial codistribution between the rates of wasting, stunting and underweight controlling for the effect of other risk factors among children under the age of 5 years in Somalia from 2007 to 2010 using the Bayesian geostatistical modelling approach.^20^ We used a shared-component model to fit common unobserved and unmeasured spatial risks to determine the areas where the indicators are strongly correlated and to identify common risk factors.

## Methods

### Survey data

The data used for this study were obtained from surveys undertaken by the Somalia Food Security and Nutrition Unit (FSNAU) and were conducted in partnership with the United Nations Children's Fund (UNICEF). The surveys were undertaken biannually from 2007 to 2010 to assess nutrition status and help with the planning of interventions.^21^ ^22^ Detailed descriptions of the survey methods and data collection are provided elsewhere.^22^ A two-stage sampling method was used with livelihoods as the first level of sampling and clusters or villages as the second level. In Somalia, livelihoods are broadly defined as agropastoral, pastoral and riverine. Pastoral communities are those that engage primarily in livestock production and are nomadic. Agropastoral communities practise mixed crop and livestock production, while those defined as riverine live along the two main rivers of the Juba and Shebelle and are primarily involved in crop production and a river-based economy.^23^ Somalia has four main seasons around which pastoral and agricultural activities depend: December to March is the ‘Jilal’ season, a harsh dry season; ‘Gu’, which is the main rainy season from April to June; from July to September is the second dry season, the ‘Hagaa’; and the short rainy season, known as ‘Deyr’, is from October to December.^24^

### Survey and environmental covariates

The covariates used in this study were related to child, household, maternal and environmental factors. At the child-level, vitamin A supplementation in the past 6 months, diarrhoea, acute respiratory infections and incidence of febrile illness in the past 2 weeks before the survey, polio and measles vaccination history, gender and age of the child were examined. At the household level, the covariates used were household size and age structure, gender of the household head, and access to different types of foods in the past 24 h. Seasonality and five environmental covariates associated with vector-borne diseases^25^ and food security^26^ were examined for modelling. These were rainfall, Enhanced Vegetation Index (EVI), mean temperature, distance to water and urbanisation. Rainfall and mean temperature were derived from the monthly average grid surfaces obtained from the WorldClim database.^27^ The EVI values were derived from the MODerate-resolution Imaging Spectroradiometer (MODIS) sensor imagery^28^ for the period 2007–2010, while the urbanisation information was obtained from the Global Rural Urban Mapping Project (GRUMP).^29^ All the environmental covariates were extracted from 1×1 km spatial resolution grids to data points. Rainfall, temperature and EVI were summarised to compute seasonal averages using the two main rainy seasons in Somalia.

### Ethical approval

Ethical approval was provided through permission by the Ministry of Health Somalia, Transitional Federal Government of Somalia Republic, Ref: MOH/WC/XA/146./07, dated 02/02/07. Owing to the high illiteracy rate of the population, informed verbal consent was sought from all participating households and individuals. An additional 10% was added to the sample size to allow for dropout or refusal to participate.

### Statistical methods

We considered three outcome measurements describing the anthropometric indicators of malnutrition: low weight-for-height (wasting), low height-for-age (stunting), and low weight-for-age (underweight) as defined in the WHO 2006 references.^30^ A child was defined as wasted, stunted or underweight when he/she was below −2 Z scores.^30^

Our analysis used joint modelling techniques for multiple health conditions^31–33^ to investigate the geographical codistribution of wasting, stunting and underweight and compute the shared correlates. The Integrated Nested Laplace Approximation (INLA) as implemented in the R-INLA library was used to produce relative risk maps at 1×1 km spatial resolution.^31^ ^34^ In the prediction model, the main survey and environmental correlates of malnutrition were controlled at the individual, household and community level. In this approach, the relative risk of each condition is assumed to depend on these correlates and an underlying geographical risk shared by the three forms of malnutrition that contribute to overall risk, in addition to condition-specific risk elements.^31^ We modelled three underlying spatial risks common to the three pairs of conditions (wasting and stunting; stunting and underweight; wasting and underweight). We also computed the degree of uncertainty in the predicted prevalence based on the estimated distribution of the shared components using the quintile correction (QC) method as implemented by Bolin and Lindgren^35^ 2012. We stratified the predicted prevalence of the shared components to generate maps that assigned each pixel into one of the four classes: less than 20%; 20%–<40%; 40%–<60% and >60% based on the predicted probabilities of class membership using the QC method.^35^ A detailed description of the methods and additional outputs from this analysis can be found in the online supplementary information (SI.1).

10.1136/bmjopen-2015-009854.supp1 Supplementary data

## Results

A total of 73 778 children under the age of 5 years were examined from 1066 clusters, of which 15 735 (21%), 22 739 (31%) and 42 271 (58%) were wasted, stunted and underweight, respectively. A total of 6640 (9%) children were wasted and stunted; 21 396 (29%) children were stunted and underweight and 14 756 (20%) were wasted and underweight. Fifty-two per cent of children were boys and the mean age of the children was 33 months. Other characteristics of the children measured during the surveys are shown in table 1. By livelihood zones, 42%, 27% and 16% of children were from areas of agropastoral, pastoral and riverine livelihoods, respectively, while 11% lived in internally displaced people (IDP) camps and 4% lived in urban areas.

**Table 1:** Summary of survey data aggregated for the period 2007–2010 (FSNAU 2007–2010)

Characteristic	Number					
Total number of children examined	73 778					
Total number of clusters examined	1066					
	Wasted; n=15 735 (21)	Not wasted; n=58 043 (79)	Stunted; n=22 739 (31)	Not stunted; n=51 039 (69)	Underweight; n=42 791 (58)	Not underweight n=30 987 (42)
Child data	Number (%)		Number (%)		Number (%)	
Vitamin A supplementation	8995 (57)	33 356 (57)	12 853 (57)	29 498 (58)	23 771 (56)	17 996 (58)
Measles vaccination	8367 (53)	30 875 (53)	11 919 (52)	27 317 (54)	21 761 (51)	16 739 (54)
Polio vaccination	12 346 (78)	46 519 (80)	18 272 (80)	40 647 (80)	33 315 (78)	24 964 (81)
Diarrhoea in the past 2 weeks	3749 (24)	10 674 (18)	5136 (23)	9159 (18)	10 280 (24)	5475 (18)
Acute respiratory infection	3797 (24)	12 029 (21)	5090 (22)	10 647 (21)	9918 (23)	6400 (21)
Febrile illness in the past 2 weeks	3793 (24)	11 912 (21)	4849 (21)	10 753 (21)	9483 (22)	6400 (21)
Suspected measles in the past 1 month	788 (5)	2487 (4)	1026 (5)	2229 (4)	1901(4)	1363 (4)
Sex of the child	Male=9039 (57)	Male=28 761 (50)	Male=12 776 (56)	Male=24 844 (49)	Male=23 888 (56)	Male=15 958 (51)
Age of the child (in months)	Mean=33, range=(6–59)	Mean=33, range=(6–59)	Mean=31, range=(6–59)	Mean=32, range=(6–59)	Mean=32, range=(6–59)	Mean=33, range=(6–59)
Age of the mother (in years)	Mean=30, range=(15–60)	Mean=30, range=(15–60)	Mean=30, range=(15–60)	Mean=30, range=(15–60)	Mean=30, range=(14–60)	Mean=30, range=(15–60)
MUAC of mother in cm	Mean=21, range=(18–38)	Mean=22, range=(18–38)	Mean=22, range=(18–38)	Mean=22, range=(18–38)	Mean=22, range=(18–38)	Mean=22, range=(18–38)
Food access data	Number (%)		Number (%)		Number (%)	
High carbohydrate foods in the past 24 h	15 227 (97)	56 059 (97)	22 285 (98)	49 008 (96)	49 009 (96)	30 023 (97)
High protein foods in the past 24 h	13 420 (85)	50 543 (87)	19 691 (87),	44 322 (87)	36 098 (84),	27 151 (88)
Fats in the past 24 h	12 327 (78)	46 277 (80)	17 751 (78),	40 880 (80)	32 422 (76),	25 040 (81)
Fruits and vegetables in the past 24 h	6423 (41)	24 835 (43)	10 695 (47)	20 665 (40)	18 492 (43)	13 068 (42)
Household data	Mean (range)		Mean (range)		Mean (range)	
Household size	6 (2–50)		6 (2–50)		6 (2–50)	
Number of under 5	2 (1–5)		2 (1–5)		2 (1–7)	
Household head gender	Male=60 128 (81%)		Male=60 128 (81%)		Male=60 128 (81%)	
Cluster data	Mean (range)		Mean (range)		Mean (range)	
Distance to water to major water bodies (km)	97 (0–508)		97 (0–508)		97 (0–508)	
Enhanced Vegetation Index (EVI)	0.18 (0–0.45)		0.18 (0–0.45)		0.18 (0–0.45)	
Precipitation (mm/year)	138 (0–350)		138 (0–350)		138 (0–350)	
Mean temperature (°C)	28 (21–31)		28 (21–31)		28 (21–31)	
Urbanisation	Urban=3318 (5%), rural=70 460		Urban=3318 (5%), rural=70 460		Urban=3318 (5%), rural=70 460	
Season	April to June (*Gu*)=47 327 (64%), October to November (*Deyr*)=26 451 (36%)		April to June (*Gu*)=47 327 (64%), October to November (*Deyr*)=26 451 (36%)		April to June (*Gu*)=47 327 (64%), October to November (*Deyr*)=26 451 (36%)	

*Gu* is the long rainy season and *Deyr* is the short rainy season from October to December in Somalia. Values in parentheses, next to the number of children, are percentages.

MUAC, mid-upper arm circumference.

The analysis of the correlates of malnutrition from the shared component model (table 2) shows that male gender was associated with a higher risk of all the three indicators of malnutrition (OR=0.75, 95% credible interval (CrI): 0.72–0.79; OR=0.75, 95% CrI: 0.72–0.79; OR=0.83, 95% CrI: 0.79–0.87 for wasting, stunting and underweight, respectively). Age 24 months or more was associated with decreased risk of wasting (OR=0.80, 95% CrI: 0.73–0.87) and increased risk of stunting (OR=1.76, 95% CrI: 1.61–1.91) and underweight (OR=1.99, 95% CrI: 1.84–2.16). The risk of stunting was higher in children in the 12–24 months age group compared to the 24–59 months age group with 6–11 months as the reference group. Children who had diarrhoea and acute respiratory infection in the past 2 weeks had a higher risk of wasting (OR=1.30, 95% CrI: 1.22–1.38; OR=1.10, 95% CrI: 1.04–1.17), stunting (OR=1.24, 95% CrI: 1.18–1.31; OR=1.21, 95% CrI: 1.15–1.27) and underweight (OR=1.26, 95% CrI: 1.17–1.35; OR=1.08, 95% CrI: 1.01–1.15). Polio vaccination was found to be associated with decreased risk of wasting but increased risk of stunting and underweight (OR=0.88, 95% CrI: 0.82–0.95; OR=1.07, 95% CrI: 1.00–1.14; OR=1.09, 95% CrI: 1.01–1.18), respectively. Increase in age of the mother was associated with decreased rates of wasting, stunting and underweight until at the age of 40 years where further increase in age did not show significant association.

**Table 2:** Multivariate adjusted OR (AOR) and 95% credible interval (CrI) of wasting, stunting and underweight among children aged 6–59 months in Somalia

	Wasting		Stunting		Underweight	
Correlates	OR	CrI	OR	CrI	OR	CrI
Child data						
Vitamin A supplementation	**0.80**	**(0.75, 0.85)**	1.00	(0.94, 1.05)	1.01	(0.94, 1.08)
Measles vaccination	1.04	(0.97, 1.11)	0.96	(0.91, 1.02)	0.93	(0.87, 1.00)
Polio vaccination	**0.88**	**(0.82, 0.95)**	**1.07**	**(1.00, 1.14)**	**1.09**	**(1.01, 1.18)**
Diarrhoea	**1.30**	**(1.22, 1.38)**	**1.24**	**(1.18, 1.31)**	**1.26**	**(1.17, 1.35)**
Acute respiratory infection (ARI)	**1.10**	**(1.04, 1.17)**	**1.21**	**(1.15, 1.27)**	**1.08**	**(1.01, 1.15)**
Febrile illness	**1.15**	**(1.09, 1.22)**	0.98	(0.93, 1.03)	1.02	(0.95, 1.09)
Suspected measles	1.05	(0.93, 1.18)	0.97	(0.87, 1.07)	0.98	(0.86, 1.11)
Sex of the child (Female)	**0.75**	**(0.72, 0.79)**	**0.75**	**(0.72, 0.79)**	**0.83**	**(0.79, 0.87)**
Child age (<12 months as reference)						
12–<24 months	**0.80**	**(0.73, 0.88)**	**2.35**	**(2.15, 2.57)**	1.05	(0.96, 1.14)
24–59 months	**0.80**	**(0.73, 0.87)**	**1.76**	**(1.61, 1.91)**	**1.99**	**(1.84, 2.16)**
Age of the mother (20–30 years as reference)						
<20 years	**1.24**	**(1.09, 1.42)**	**1.04**	**(1.02, 1.06)**	**1.08**	**(1.01, 1.16)**
31–40 years	**0.91**	**(0.83, 0.99)**	**0.87**	**(0.83, 0.91)**	**0.94**	**(0.93, 0.95)**
41–50	**0.88**	**(0.83, 0.93)**	**0.91**	**(0.84, 0.99)**	1.01	(0.88, 1.15)
>50 years	0.92	(0.85, 1.01)	0.76	(0.49, 1.17)	1.01	(0.49, 2.08)
MUAC of mother	0.99	(0.99, 1.00)	1.00	(1.00, 1.00)	**0.99**	**(0.98, 0.99)**
Household data						
Household size	**1.14**	**(1.13, 1.15)**	**1.27**	**(1.25, 1.28)**	1.00	(0.99, 1.01)
Number of under 5	**1.06**	**(1.03, 1.09)**	**1.80**	**(1.76, 1.85)**	1.00	(0.97, 1.04)
Female household head	0.95	(0.89, 1.02)	**0.87**	**(0.82, 0.92)**	0.98	(0.92, 1.05)
Food access data						
High-carbohydrate foods	**0.83**	**(0.79, 0.88)**	**0.82**	**(0.78, 0.86)**	**0.92**	**(0.87, 0.97)**
High-protein foods	**0.57**	**(0.55, 0.59)**	**0.51**	**(0.49, 0.52)**	**0.90**	**(0.87, 0.93)**
Fats	1.04	(0.98, 1.12)	**0.92**	**(0.87, 0.98)**	**1.10**	**(1.02, 1.18)**
Fruits and vegetables	0.97	(0.93, 1.01)	**1.04**	**(1.01, 1.08)**	**1.07**	**(1.03, 1.12)**
Cluster data						
Season (October to November as reference)	**1.11**	**(1.04, 1.18)**	1.04	(0.98, 1.10)	**0.93**	**(0.91, 0.94)**
Distance to water	1.00	(1.00, 1.00)	1.00	(1.00, 1.00)	1.00	(1.00, 1.00)
Enhanced Vegetation Index (EVI)	**0.66**	**(0.45, 0.95)**	**0.59**	**(0.42, 0.82)**	**0.69**	**(0.67, 0.72)**
Rainfall	**0.88**	**(0.79, 0.98)**	1.09	(0.96, 1.22)	0.95	(0.86, 1.06)
Temperature	**1.07**	**(1.03, 1.11)**	**1.05**	**(1.01, 1.10)**	**1.12**	**(1.07, 1.17)**
Urbanisation	**0.96**	**(0.94, 0.99)**	1.00	(0.97, 1.04)	0.97	(0.94, 1)

The estimates were derived from joint distribution modelling and the results are indicative of the effect of the shared components.

Values in bold typeface are those that do not contain the value 1 in their 95% CrI and were considered statistically significant.

Increases in the household size and number of under-fives in the household were associated with increased risk of wasting and stunting, but not with underweight. Children who had consumed any of the staple sources of carbohydrates or proteins within the 24 h prior to the survey had a lower risk of all the three indicators of malnutrition. In addition, children who had consumed fruits and vegetables had a higher risk of stunting and underweight. Vitamin A supplementation was associated with low risk of wasting (OR=0.80, 95% CI 0.75 to 0.85) but was not associated with stunting and underweight. There was a significant association between the vegetation index (OR=0.66, 95% CrI: 0.45–0.95), (OR=0.59, 95% CrI: 0.42–0.82), (OR=0.69, 95% CrI: 0.67–0.72) and temperature (OR=1.07, 95% CrI: 1.03–1.11), (OR=1.05, 95% CrI: 1.01–1.10), (OR=1.12, 95% CrI: 1.07–1.17) with wasting, stunting and underweight, respectively. Season was associated with wasting (OR=1.11, 95% CrI: 1.04–1.18) and underweight (OR=0.93, 95% CrI: 0.91–0.94), but had no association with stunting. Urbanisation was associated with decreased risk of wasting (OR=0.96, 95% CrI: 0.94–0.99), but was not associated with stunting and underweight.

**Figure 1:**
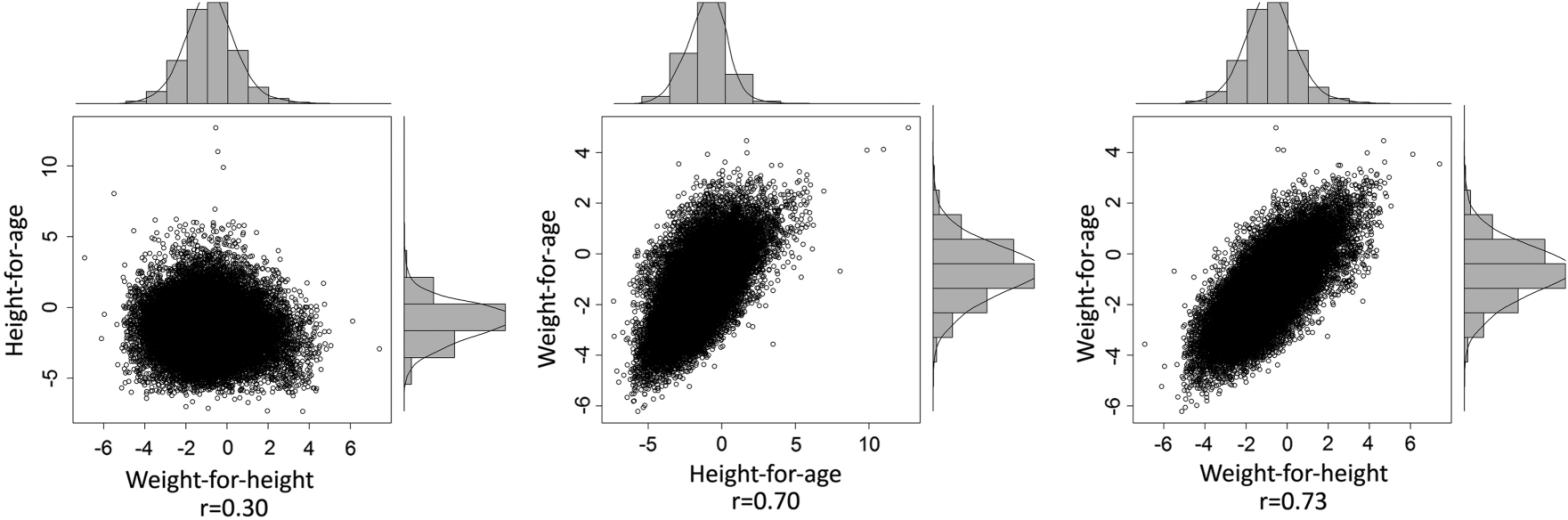
Correlation plots of weight-for-height, height-for-age and weight-for-age Z-scores among children aged 6–59 months in Somalia using survey data from 2007 to 2010.

The linear correlation between weight-for-age and weight-for-height was 0.73 and that between height-for-age and weight-for-age was 0.70 (figure 1). However, at 0.30, the correlation between weight-for-height and height-for-age was low.

**Figure 2:**
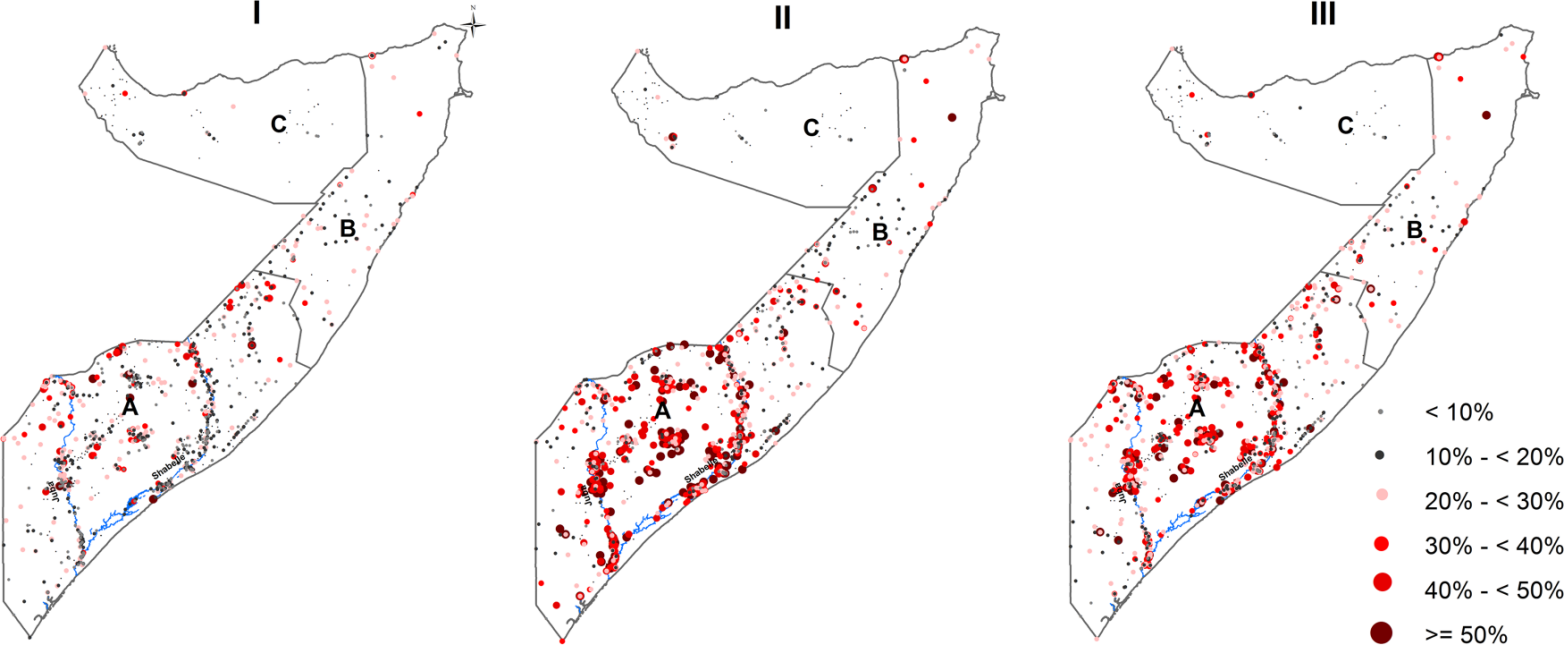
Maps showing the observed prevalence of wasting (I), stunting (II) and underweight (II) by survey cluster between 2007 and 2010 in Somalia. The country is divided into three main zones: South Central (A), North East (B) and North West (C). Seventy eight clusters were sampled in the North West zone, 85 clusters in the North East zone and 903 clusters in the South Central zone. The country's two main rivers, Juba and Shebelle, are located in the South Central zone.

**Figure 3:**
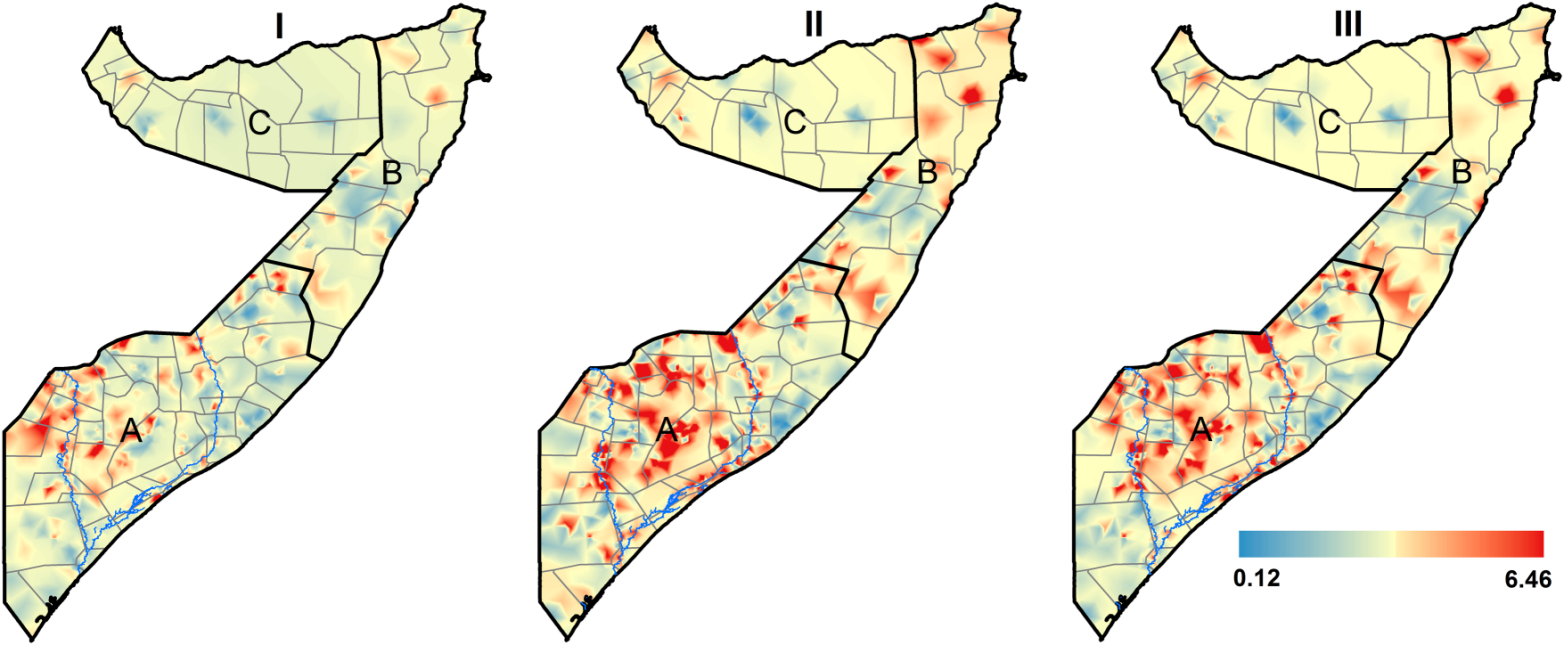
Maps of the relationships of the spatial distribution of residual relative risks common to: (I) wasting and stunting; (II) stunting and underweight; and (III) wasting and underweight among children aged 6–59 months in Somalia. Each map is plotted at 1×1 km spatial resolution. South Central (A), North East (B) and North West (C).

**Figure 4:**
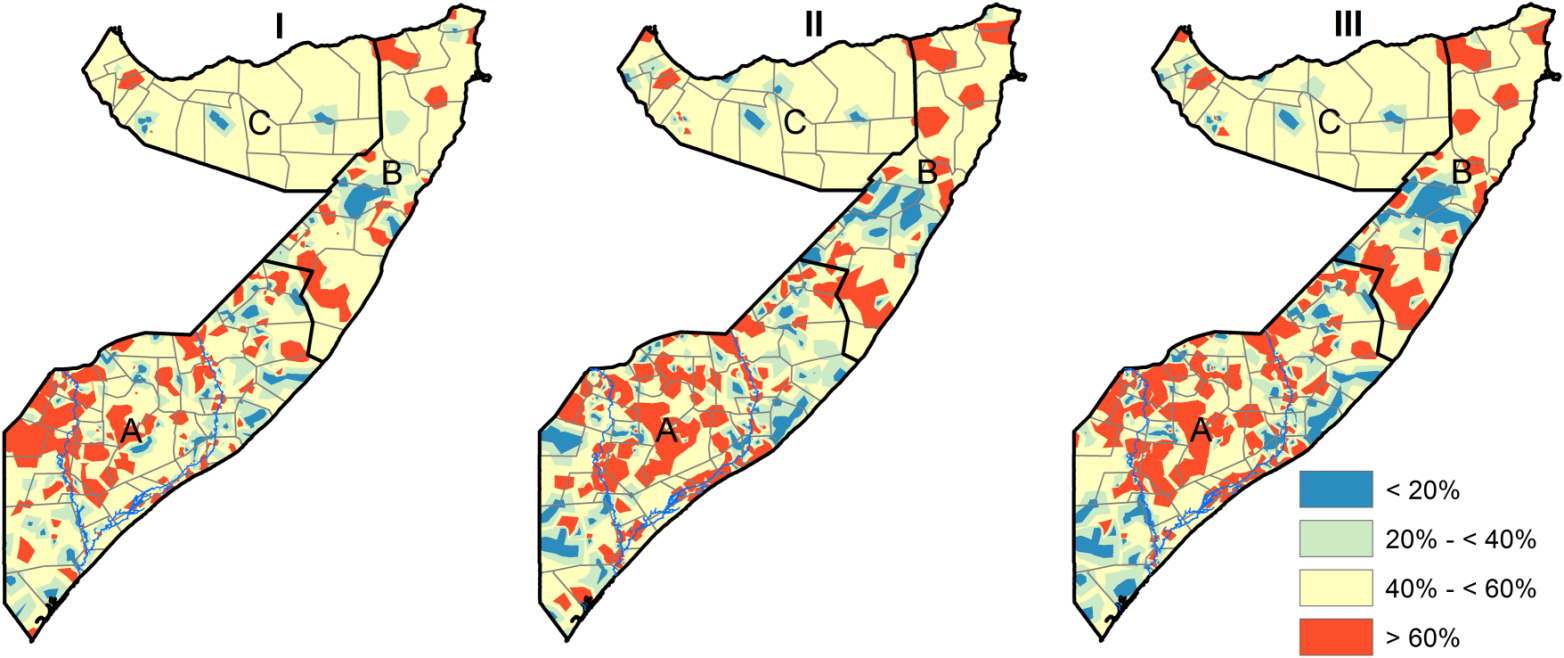
Estimated shared components classified at 95% credible level among children aged 6–59 months using the marginal probabilities calculated using the quintile correction (QC) method.^35^ I=Wasting and Stunting, II=Stunting and Underweight, III=Wasting and Underweight. South Central (A), North East (B) and North West (C).

Figure 2 shows the spatial distribution of the observed prevalence of wasting, stunting and underweight by cluster for FSNAU nutrition surveys conducted from 2007 to 2010. The relationship in spatial distributions was significant for the three pairs of indicators: (OR=2.58, 95% CrI: 1.32–5.01) for wasting and stunting; (OR=3.93, 95% CrI: 3.37–4.59) for stunting and underweight and (OR=2.04, 95% CrI: 1.92–2.17) for wasting and underweight. Estimated common spatial patterns are shown in figure 3. The maps reveal geographical variation in relative risk of the three indicators. A strong spatial gradient in the South-North direction was present in all the shared components examined in this study. This confirms a high risk of all forms of malnutrition in the southern regions, especially around the two main rivers of Juba and Shebelle, compared to the Northern regions of Somalia. The relationship in the spatial distribution was highest between stunting and underweight with relative risk values ranging between 0.15 and 6.20, followed by wasting and underweight (0.18–5.18) and lowest between wasting and stunting (0.26–4.32), with larger effects observed in the southern regions of Somalia. The risk stratified maps of the predicted prevalence are shown in figure 4. The SDs ranged from 0.04 to 0.40 (see online supplementary figure SI. 1). Summaries of the parameters used in the joint modelling for the three indicators of nutrition status are shown in online supplementary figure SI. 2.

## Discussion

We have implemented a joint spatial analysis of malnutrition among children under the age of 5 years in Somalia to identify shared and separate covariate and spatial components of wasting, stunting and underweight. The correlation between regional means of relative risks was highest between stunting and underweight, followed by wasting and underweight, while the correlation between wasting and stunting was relatively low. There were several common underlying components of the three measures that influenced the spatial codistribution of the three indicators of malnutrition in Somalia. Research and nutrition programmes have focused on wasting and stunting in accessing nutritional status, designing programmes, and assessing impact, and therefore our discussion will focus on these two indicators.^8^ ^36–38^ Access to foods high in protein and vegetation cover, a proxy of rainfall or drought, were associated with lower risk of wasting and stunting. Age, gender, illness, access to carbohydrates and temperature were also common correlates of all three indicators.

Stunting increases throughout the first 2–3 years of life in many developing countries, whereas wasting occurs during the first year of life after which it stabilises, as expected.^10^ Wasting was also noted to have a relatively shorter duration and greater seasonal variability when compared with stunting.^10^ This may explain the observed low association between wasting and stunting. Thus, the use of cross-sectional survey data to describe trends in wasting may have some limitations since the observed prevalence has short interval fluctuations and varies substantially between seasons.^39^ As a result, a high incidence of wasting of short duration might be missed, misrepresenting the relationship of wasting with the other indicators. Longitudinal studies which follow children's growth from birth looking at the wasting, stunting and the combination of the two can provide better understanding of the relationships.^11^

Food insecurity and infectious diseases differ between regions and by type of livelihood resulting from a combination of harsh environmental conditions and prolonged conflict and civil insecurity.^38^ In this study, access to food and infections were major factors associated across the three indicators of malnutrition and this reaffirms the strong shared component of wasting and stunting in the southern region relative to the northern region in Somalia.^22^ For example, there are currently about 400 000 IDPs in Somalia, mostly from the Southern minority groups. Half of this population lives in Mogadishu, almost completely out of reach of any concrete internal assistance.^22^ This is one area that had consistently strong shared components of the malnutrition indicators in this study. Kismaayo, also another consistent hotspot, has been host to numerous IDPs seeking refuge from drought and conflict in the Juba and other neighbouring regions.^40^ IDPs are considered to be among the poorest population groups in the country with high levels of food insecurity with poor living conditions that predispose them to various infectious diseases.^40^

According to 2010 estimates, agriculture accounts for 65% of the gross domestic product (GDP), with livestock representing 40% of the GDP and 65% of the export earnings in Somalia. However, farming and pastoralist livelihoods have been hindered by the long-standing civil war and the internal displacements of populations.^40^ In addition, unpredictable rainfall seasonality, anomalies and consequent droughts have played a role. In our analysis, EVI, a satellite imagery derived variable which characterises the global range of vegetation state, was a significant common factor across the indicators of malnutrition. High EVI values indicate vegetated areas that reflect forested areas, riverine vegetation and, more importantly, local agriculture which has a direct relationship with the local food security. Highly vegetated areas (as demarcated by high EVI values) are a product of a combination of several variables including rainfall, seasonal and permanent water sources and, to some extent, underground water. In arid and semiarid areas, agricultural activities are limited to areas with reliable water supply. Temperature was also found to be an important correlate of malnutrition. Temperature is directly linked to aridity,^41^ which in turn has an impact on malnutrition.^26^ High-temperature values are reported in Somalia, rising up to 40°C in the Hagaa dry season^42^ between the months of July and September. The seasonal variation in these environmental factors also affect the intra-annual variations of the burden of malnutrition, with wasting particularly sensitive to seasonality in Somalia and other places.^39^ ^43^ Seasonality affects the general availability of food and the rates of infection, but in Somalia, which is heavily dependent on pastoral and subsistence agriculture, it also undermines household income and resilience.^40^ Among the pastoralist communities in Somalia, which are predominantly in the northern regions, the seasonal migration in search of water and pasture for their livestock has been a mechanism of mitigating the adverse effects of droughts and may contribute to the spatial variability of hotspots from year to year.^40^

Our findings suggest that integrated programming and interventions focused on the common risk factors of the three indicators and, specifically in regions where the codistribution is highly prevalent, may be a more effective way of reducing the burden of malnutrition in Somalia. Currently, however, the funding for the nutrition programme in Somalia is limited, unstable and often short term, preventing investment in longer term sustainable and resilience-building programmes.^38^ As a result, for the large part, humanitarian organisations are obliged to focus on high impact, ‘life-saving’ interventions to treat acute malnutrition without opportunities to invest in preventative programmes to reduce the overall caseloads and risk of undernutrition at scale.^38^ Much of this is due to the focus of response in Somalia being emergency-driven. However, the information provided by this study on the common drivers and the extent of geographical coexistence of wasting and stunting can be used to develop more informed and planned interventions to achieve maximum impact within the short term and available funding.

Information generated from this study could also help in the development of an improved nutrition surveillance system with sensitive indicators of the different forms of undernutrition. This would include modifying data collection tools to reflect the main drivers of malnutrition and strategically position surveillance centres in regions that would provide the right information for intervention at the right time to inform the most appropriate response and maximise impact and investment.^44^

The consistency and timeliness of FSNAU survey data provide an opportunity to analyse the trends of malnutrition in Somalia. The use of individual level data has improved the estimates in our analysis as it accounted for the variability between children ensuring more accurate estimates than use of aggregated data. However, the use of the WHO reference population may have limitations in some populations. It has been previously noted that children in Somalia have lower weight at birth and by 12–24 months these children are thinner and taller with half the stunting prevalence when compared to other children in the neighbouring East African countries.^45^ Pastoral children's growth patterns may differ from those of children in populations with agricultural livelihoods, for example.^46^ The references may therefore underestimate malnutrition in some areas. The effect of conflict on malnutrition was not controlled in this study because the information was not captured during the FSNAU surveys. Further research should include conflict, and longitudinal studies should be undertaken to provide a better understanding of the relationship between wasting and stunting.

## Conclusion

This study has demonstrated that wasting, stunting and underweight in children aged 6–69 months in Somalia share common risk factors with evidence of correlation in space.^17^ ^47^ The emergency response funding is by nature short term the of spatial patterns and trends of wasting and stunting and information on seasonal variation and the age and gender of the child can be used to support effective interventions. Although emergency nutrition response in Somalia focuses on wasting, our evidence suggests that implementation of a more joined-up programme may be most effecive. This will require political will, appropriate financing, policies and programmatic links between partners on the main indicators of malnutrition.
