# Supplementary material for: Assessing comorbidity and correlates of wasting and stunting among children in Somalia using cross-sectional household surveys: 2007 to 2010
Source: BMJ Open. 2016 Mar 9;6(3):e009854. doi: 10.1136/bmjopen-2015-009854 (PMC4785320; doi:10.1136/bmjopen-2015-009854)
Supplement: Supplementary data [file bmjopen-2015-009854supp.pdf]

Supplementary Information: Model procedures and model output

SI.1 Space-time Bayesian Geo-statistical shared component model

Disease mapping mainly focuses on modelling single disease yet many diseases share common risk factors[1]. This underscores the importance of joint analysis of health conditions to identify similar patterns in geographical variation and provide more substantial evidence on clustering of the underlying risk surface for integrated interventions[2]. Non-Bayesian multilevel models have been used to present joint spatial analyses of diseases[3]. Knor-Held suggested joint modelling approaches in Bayesian perspective as an improvement of Besag *et al* formulations[4]. Shared spatial component methods have been used extensively to jointly model risk of more than one disease outcome[2 5 6]. Lindgren et al 2013 suggested that one can express a large class of random field models as a solution to continuous domain stochastic partial differential equations (SPDEs), and develop explicit links between the parameters of each SPDE and the elements of precision matrices for the weights in a discrete basis function representation[7]. This SPDE is formulated as a link between Gaussian random fields (GRFs) and the Gaussian Markov Random Fields (GMRFs)[7].

In this study, we implemented the Bayesian geo-statistical shared component model of three nutritional indicators through SPDE approach using R-INLA library[7]. Suppose using logit model for wasting, stunting and underweight, effect of covariates and latent spatial effect,

$$\log it(p_{i1}) = \alpha_1 + S_{i1} \quad (1)$$

$$\log it(p_{i2}) = \alpha_2 + S_{i2} \quad (2)$$

$$\log it(p_{i3}) = \alpha_3 + S_{i3} \quad (3)$$

This is where p_{i1}, p_{i2}, p_{i3} were the relative risks for wasting, stunting and underweight in cluster i respectively. The parameter α_j is the disease-specific intercept, β_j s are the indicator specific risk coefficients associated with the risk vector x; and S is the latent spatial effect. The spatial effects in this model can be expressed as:

$$S_{1i} = u_{1i} + \beta_{12} u_{2i} \quad (4)$$

$$S_{2i} = u_{2i} + \beta_{23} u_{3i} \quad (5)$$

$$S_3 = u_3 + \beta_{31} u_{1i} \quad (6)$$

This is where u_{1i} is the shared component for wasting and stunting; u_{2i} is the shared component for stunting and underweight; u_{3i} is the shared component for wasting and underweight. β_{12}, β_{23} and β_{31} are the covariates effects of the shared components of wasting and stunting; stunting and underweight and wasting and underweight respectively[2 5].

References

1. Held L, Natario I, Fenton SE, et al. Towards joint disease mapping. Statistical methods in medical research 2005;**14**(1):61-82.
2. Manda SM, Feltbower RG, Gilthorpe MS. Review and empirical comparison of joint mapping of multiple diseases. Southern African Journal of Epidemiology and Infection 2011;**27**(4):169-82.
3. Langford IH, Bentham G, McDonald A-L. Multi-level modelling of geographically aggregated health data: a case study on malignant melanoma mortality and UV exposure in the European Community. Statistics in Medicine 1998;**17**(1):41-57.
4. Besag J, Green PJ. Spatial statistics and Bayesian computation. Journal of the Royal Statistical Society Series B (Methodological) 1993.
5. Held L, Graziano G, Frank C, et al. Joint spatial analysis of gastrointestinal infectious diseases. Statistical methods in medical research 2006;**15**(5):465-80.
6. Kandala N-B, Manda SO, Tigbe WW, et al. Geographic distribution of cardiovascular comorbidities in South Africa: a national cross-sectional analysis. Journal of Applied Statistics 2013(ahead-of-print):1-14.
7. Lindgren F, Rue H. Bayesian spatial and spatiotemporal modelling with r-inla. Journal of Statistical Software 2013.

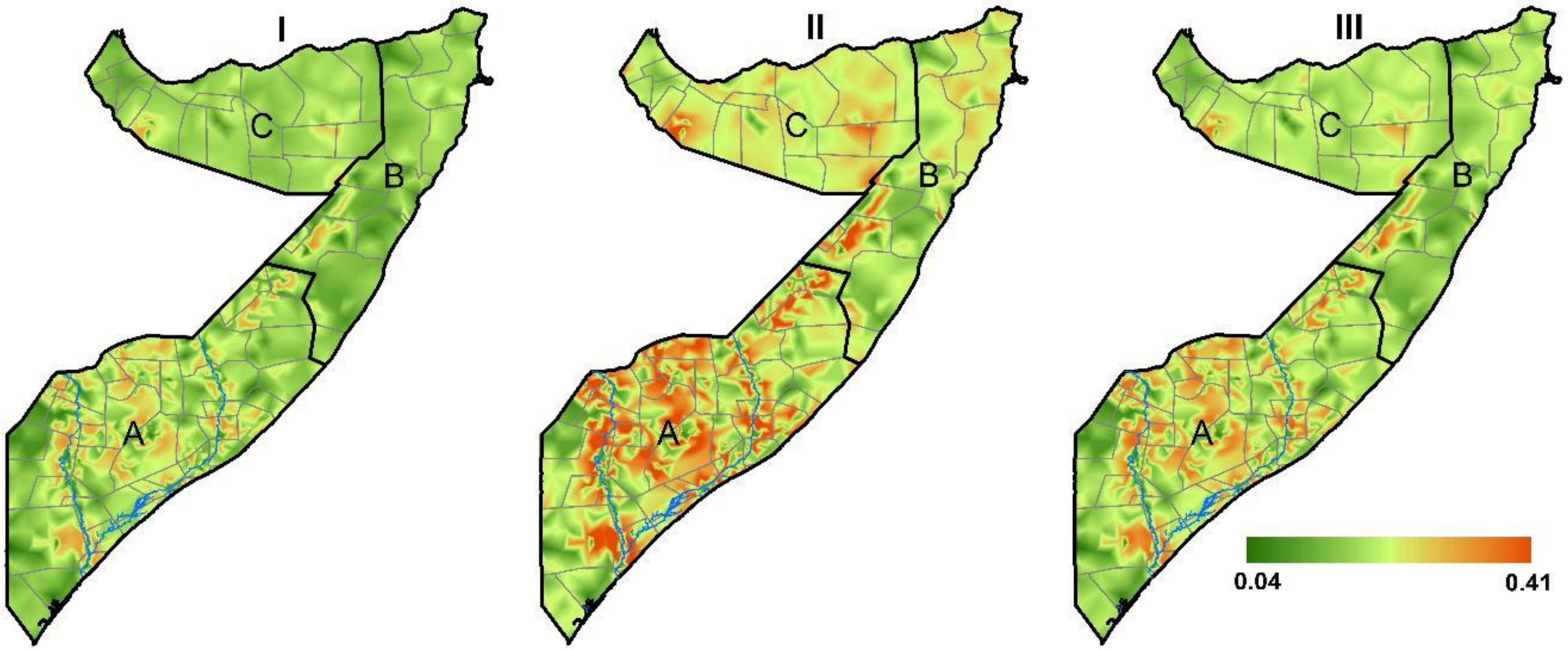

Figure SI. 1: Coefficients of variation at 1 x 1 km spatial resolution among children aged 6 - 59 months in Somalia. I=Wasting and Stunting, II=Stunting and Underweight, III=Wasting and Underweight. A=South-central zone, B=Northeast (Puntland) zone, C=Northwest (Somaliland) zone. 1=Wasting, 2=Stunting, 3=Underweight.

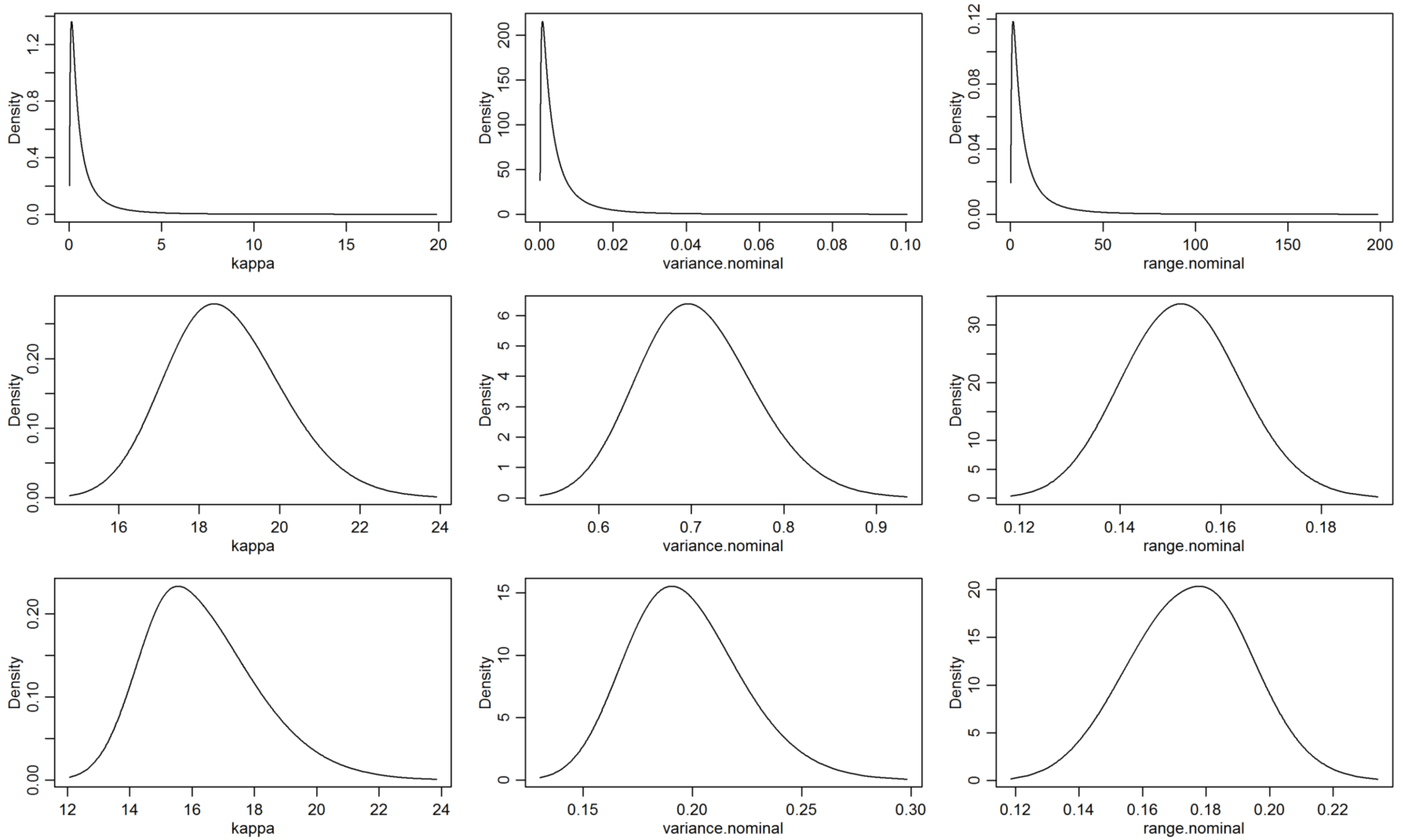

Figure SI. 2: Summary of the posterior marginal for kappa, variance and range of the three fields for the wasting, stunting and underweight.
